# Constraint and cost of oxidative stress on reproduction: correlative evidence in laboratory mice and review of the literature

**DOI:** 10.1186/1742-9994-9-37

**Published:** 2012-12-26

**Authors:** Antoine Stier, Sophie Reichert, Sylvie Massemin, Pierre Bize, François Criscuolo

**Affiliations:** 1 Centre National de la Recherche Scientifique, UMR7178, Strasbourg, 67037, France; 2 Université de Strasbourg, IPHC, 23 rue Becquerel, Strasbourg, 67087, France; 3 Department of Ecology and Evolution, University of Lausanne, Biophore, Lausanne-Dorigny, 1015, Switzerland

**Keywords:** Life-history trade-offs, Reactive oxygen species, Antioxidant, Ageing, Literature review

## Abstract

**Background:**

One central concept in evolutionary ecology is that current and residual reproductive values are negatively linked by the so-called cost of reproduction. Previous studies examining the nature of this cost suggested a possible involvement of oxidative stress resulting from the imbalance between pro- and anti-oxidant processes. Still, data remain conflictory probably because, although oxidative damage increases during reproduction, high systemic levels of oxidative stress might also constrain parental investment in reproduction. Here, we investigated variation in oxidative balance (*i*.*e*. oxidative damage and antioxidant defences) over the course of reproduction by comparing female laboratory mice rearing or not pups.

**Results:**

A significant increase in oxidative damage over time was only observed in females caring for offspring, whereas antioxidant defences increased over time regardless of reproductive status. Interestingly, oxidative damage measured prior to reproduction was negatively associated with litter size at birth (constraint), whereas damage measured after reproduction was positively related to litter size at weaning (cost).

**Conclusions:**

Globally, our correlative results and the review of literature describing the links between reproduction and oxidative stress underline the importance of timing/dynamics when studying and interpreting oxidative balance in relation to reproduction. Our study highlights the duality (constraint and cost) of oxidative stress in life-history trade-offs, thus supporting the theory that oxidative stress plays a key role in life-history evolution.

## Introduction

A central concept in evolutionary ecology is that fitness-related traits, which allow organisms to produce many offspring over many reproductive attempts (i.e. fecundity and survival), are traded-off amongst themselves due to a ubiquitous constraint: a limited available pool of resources to share between all biological functions [1]. Hence, current parental investment in reproduction is expected to result in decreased survival and future reproductive value known as the so-called cost of reproduction [1,2]. It has been established for some time that life-history trade-offs are the bedrock of evolutionary biology, and in recent years attention has turned towards examining the nature of the mechanisms underlying trade-offs. In this context, the production of reactive oxygen species (ROS), by-products of oxidative metabolism, appears to be a corner-stone factor both by its universal and inevitable nature [3].

Oxidative stress is defined as an imbalanced situation where deleterious production of ROS (mainly by the mitochondria during normal energy processing) exceeds the capacity of the various anti-oxidant systems to deal with them [4]. The balance between pro-and anti-oxidative processes determines the level of oxidative stress: the higher the production of ROS and/or the lower the antioxidant defences are, the higher the oxidative stress will be. This oxidative imbalance is known to cause damage to all types of biomolecules, and the accumulation of damage over time is thought to contribute to ageing [4,5]. Thus, if we postulate that the cost of reproduction is mediated by mechanisms having antagonistic effects on fecundity and survival [6], measuring the variation of a pro-ageing mechanism such as oxidative stress might provide important mechanistic insights into the cost of reproduction.

Following this idea, two innovative studies performed in captive zebra finches (*Taeniopygia guttata*) indicate oxidative stress as a proximate mechanism for the cost of reproduction [7,8]. Indeed, parents forced to rear extra offspring were seen to down-regulate important antioxidant enzymes (SOD, GPx, [8]) and were less capable of dealing with oxidative stress, measured as a decline in the resistance of red blood cell membranes to an oxidative burst [7]. Because oxidative damage can impede biological function, including reproduction, previous studies have also proposed that oxidative stress can act as a constraint, limiting parental investment in reproduction [9,10]. This idea is also supported by toxicological studies showing that pollution or experimental contamination are often associated with higher levels of oxidative stress and impaired reproductive capacities (reviewed by [3], see also [11] for evidence from biomedical research on fertility). Such a constraining effect of oxidative stress was recently illustrated by a cross fostering experiment in a wild population of Alpine swifts, where egg hatching success was positively related to the resistance of red blood cell membranes to oxidative burst in their biological rather than their foster mothers [9]. However, as adult females in this study were blood sampled after egg laying, the impact of pre-reproductive oxidative stress levels on current adult reproduction output on one hand (i.e. constraint), and the level of oxidative stress induced as a result of current reproduction (i.e. cost) on the other remains to be tested within a single longitudinal study. Finally, the three aforementioned studies [7-9] were based on measurements of antioxidant status or cell resistance to oxidative burst, but for a full understanding, studies on oxidative stress should ideally include at least two sides of the oxidative balance (i.e. ROS production, defences or the resulting damage) [12]. These discrepancies in measurements are important and join the contrasted recent results on the oxidative cost of reproduction, showing either weak [13] or inconclusive effects of reproduction on the oxidative balance [14-16]. All these studies point to the importance of the timing (pre- *versus* post- reproduction) and the choice of the markers (defence *versus* damage) in the investigation of the links between oxidative stress and reproduction. Indeed, while oxidative damage can worsen during reproduction, high oxidative stress levels in pre-reproductive adults might also act as a constraint [10], limiting individual investment in reproduction. Hence, cost and constraint on reproduction may finally explain why oxidative stress and reproduction are sometimes positively or negatively linked together.

Our aim was to address this issue by investigating the possible dual role of oxidative stress as both a constraint and a cost of reproduction. To this end, we measured both sides of the oxidative balance (*i*.*e*. oxidative damage and antioxidant defences) before and after reproduction of female laboratory mice, and investigated how litter size at birth and at weaning related to measurements of the oxidative balance before and after reproduction, respectively. We predicted that if oxidative stress acts as a constraint on reproduction, measurements of oxidative balance before reproduction should be negatively related to the reproductive output. Furthermore, if reproduction induces oxidative stress, we expected positive relations between the reproductive output and measurements of oxidative balance after reproduction.

## Results

### Variation of oxidative balance over the course of reproduction

**Figure 1:**
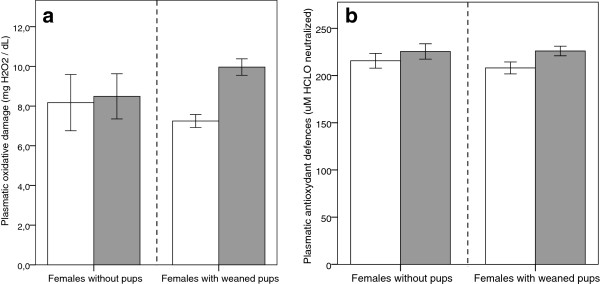
**Female mice plasmatic oxidative balance parameters (n = 23), measured before (white bars) and 18 days after reproduction (grey bars).** Data are presented for female mice that succeeded to reproduce (N = 18; right-hand side of figure) and those that did not (N = 5; left-hand side of figure) (see Methods for details)**. (a)** Mean ± SE oxidative damage (d-ROMs) increased only in successful females. **(b)** Mean ± SE plasmatic antioxidant barrier (OXY) as a global marker of antioxidant defences, rises similarly throughout the study in both groups (see text for statistics).

Changes over time in the plasmatic oxidative damage of successful and unsuccessful females were significantly explained by the interaction between reproductive status and reproductive period (*p* = 0.009; Table 1). Indeed, the comparison of d-ROM levels before and after a successful reproduction revealed a significant increase (37.5%) in oxidative damage (Figure 1a; paired t-test, *t*_paired_ = −6.53, df = 17, *p* < 0.001), whilst these levels were not observed to differ from each other in females without offspring’s (Figure 1a; t_paired_ = 0.68, df = 4, *p* = 0.54). Variation in plasmatic antioxidant defences showed a moderate but significant increase over time of 6.6% (reproductive period: p = 0.05; Table 1, Figure 1b). However, this increase in antioxidant defences was not influenced by reproductive status alone (p = 0.72), or by the interaction between reproductive period and reproductive status (p = 0.55; Table 1, Figure 1b). Successful and unsuccessful females did not significantly differ in oxidative balance parameters before reproduction. Indeed, d-ROM levels (7.25 ± 0.33 *vs*. 8.17 ± 1.42; t-test, t = 0.98, df = 21, *p* = 0.34) and OXY levels (208.1 ± 6.3 *vs*. 215.6 ± 7.8; t-test, t = 0.59, df = 21, *p* = 0.56) were not statistically different between those two groups.

**Table 1:** Results of mixed model showing the effect of reproductive status (successful vs. unsuccessful) and reproductive period (before or during reproduction) on plasmatic oxidative damage (d-ROMs) and plasmatic antioxidant defences (OXY) levels in female mice

**d-ROMs**	**Random Effects**	**Estimate**	**SE**		
	Constant	2.24	0.93		
	Individual	1.37	0.42		
	**Fixed Effects**	**Estimate**	**SE**	**F1,21**	**P value**
	Intercept	9.97	0.48		
	Reproductive Status	−1.48	0.96	0.10	0.753
	**Reproductive Period**	**−2.72**	**0.39**	**13.17**	**0.002**
	**Status × Period**	**2.1**	**0.84**	**8.25**	**0.009**
**OXY**	**Random Effects**	**Estimate**	**SE**		
	Constant	187.9	124.5		
	Individual	351	108.3		
	**Fixed Effects**	**Estimate**	**SE**	**F1,21**	**P value**
	Intercept	226	5.47		
	Reproductive Status	−0.49	11.74	0.14	0.716
	**Reproductive Period**	**−17.94**	**6.25**	**4.31**	**0.050**
	Status × Period	8.08	13.4	0.36	0.553

Significant terms are reported in boldface. Non-significant interactions were backward dropped from the model. Residuals of the models follow a normal distribution, all Kolmogorov-Smirnov tests, *P* > 0.79.

### Correlations between reproductive and oxidative parameters

**Figure 2:**
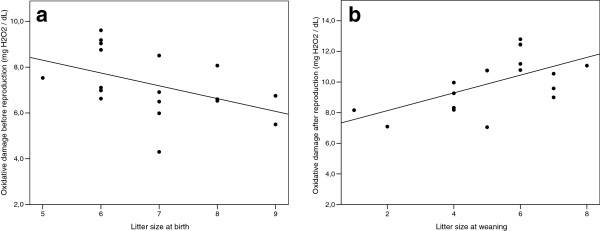
**Relationships between litter size and oxidative damage (n = 18 in both cases). (a)** Pre-reproductive oxidative damage (d-ROMs) in relation to litter size at birth (*p* = 0.039), **(b)** Post-reproductive oxidative damage (d-ROMs) in relation to litter size at weaning (*p* = 0.006). Unsuccessful females are not represented here because they either did not reproduce or they may have failed early in reproduction for other reasons than initial elevated oxidative stress levels. Notice that overlapping values are reducing to 16 the number of readily apparent points.

**Figure 3:**
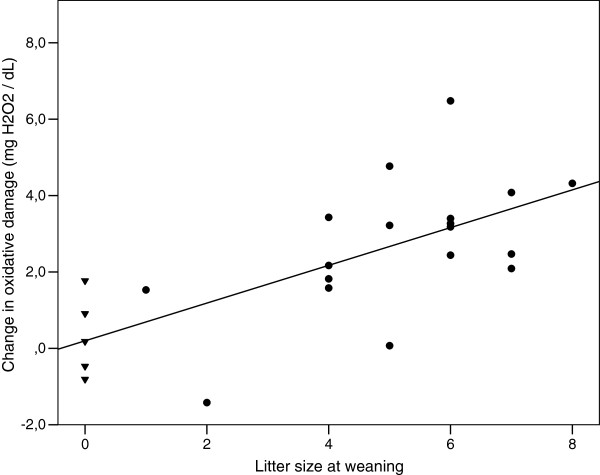
**Female reproductive output and changes in oxidative damage during reproduction (n = 23).** Change in plasmatic oxidative damage (d-ROMs) during reproduction is positively related to litter size recorded at weaning (ANCOVA, F = 19.81, df = 1, *p* <0.001). Successful females (black dots) and unsuccessful females (black triangle) are shown but the relationship remains significant without unsuccessful females (ANCOVA, F = 7.24, df = 1, *p* = 0.016).

Before reproduction, oxidative damage (but not antioxidant defences) was negatively related to litter size at birth (Table 2). Less offspring were born to successful females in which relatively high oxidative damage levels had been seen initially (Figure 2a). After reproduction, the relation between d-ROMs levels and litter size was inverted, with larger litter sizes at weaning being associated with higher oxidative damage in post-weaning females (Table 2, Figure 2b). Again, antioxidant defence levels were not related to litter size at weaning (Table 2). Models testing litter size 1) at weaning in relation to d-ROMs or OXY levels before reproduction and 2) at birth in relation to d-ROMs or OXY levels after reproduction, showed no significant effects (Table 2). Finally, models testing whether intra-individual changes in oxidative damage and antioxidant defences over the course of reproduction were related to reproductive output evidenced that females producing the greatest number of pups at weaning were also those suffering the higher increase in oxidative damage (Figure 3, ANCOVA, F = 19.81, df = 1, *p* <0.001). No such relationship was found with plasmatic changes of antioxidant defences (ANCOVA, F = 0.22, df = 1, *p* = 0.65).

**Table 2:** Analyses of covariance on relationships between pre (1) and post-reproductive (2) d-ROMs and OXY plasma levels and litter size at birth and at weaning in female mice

**Litter size at birth**	**Pre-reproductive values**	**df**	**Estimate**	**SE**	**Khi2**	**P-value**
	Intercept		8.08	2.21		
	**d-ROMs(1)**	**1,17**	**−0.37**	**0.17**	**4.25**	**0.039**
	OXY(1)	1,17	0.01	0.01	0.65	0.419
	**Post-reproductive values**					
	Intercept		6.04	3.62		
	d-ROMs (2)	1,17	−0.08	0.16	−0.51	0.621
	OXY(2)	1,17	0.01	0.011	0.55	0.588
**Litter size at weaning**	**Pre-reproductive values**					
	Intercept		6.27	4.36		
	d-ROMs(1)	1,17	0.05	0.33	0.14	0.893
	OXY(1)	1,17	−0.01	0.02	−0.40	0.694
	**Post-reproductive values**					
	Intercept		0.60	4.30		
	**d-ROMs (2)**	**1,17**	**0.59**	**0.19**	**7.44**	**0.006**
	OXY(2)	1,17	−0.01	0.02	0.13	0.716

Significant terms are reported in boldface. Residuals of each model follow a normal distribution, all Kolmogorov-Smirnov tests, *P* > 0.62.

## Discussion

In the present study, we show that pre-reproductive oxidative damage levels in female laboratory mice were negatively related to litter size at birth, whereas post-reproductive damage levels were positively related to litter size at weaning. Before mating, there was no difference in terms of age, mass and oxidative stress levels between females that then produced successfully or not a litter. However, oxidative damage levels increased strongly (+35%) over time in successfully reproducing females and remained stable in unsuccessful ones during the same time window. It suggests that reproduction (gestation and/or lactation) can lead to oxidative damage in female mice.

### Oxidative stress as a cost of reproduction

Oxidative stress is due to an imbalance between ROS production and the antioxidant system [4]. Hence, an oxidative reproductive cost can occur if reproduction is associated with an increase of the production of ROS, a down-regulation of the antioxidant defences, or a combination of those two processes [12]. In the present study, plasmatic antioxidant levels do not seem to be affected by reproduction: there was no difference in antioxidant defences between female mice that reproduced and those that did not, and changes in antioxidant defences during reproduction did not correlate with the number of offspring weaned. Hence, one hypothesis is that the observed increase in plasmatic oxidative damage associated to reproduction in female mice is most probably linked to an increase in ROS production. This oxidative cost of reproduction seemed to vary with reproductive investment, as suggested by the positive relationship between litter size at weaning and oxidative damage after reproduction and the increase in intra-individual damage with litter size at weaning. Although the increase in metabolic demands with reproduction has already been acknowledged [17,18], the link between metabolic rate and ROS production is still controversial [19]. More work is therefore required to firmly establish a chain of causation between reproduction, metabolic demands, ROS production and ultimately oxidative damage. A second hypothesis previously suggested by Wiersma et al. [8] is that parents ‘sacrifice oxidative protection for reproduction’, thereby leading to oxidative stress even in the absence of any increase in metabolic demands and ROS production. Although we found no significant support for this hypothesis in terms of plasmatic antioxidant defences (but our limited sample size does not allow firm conclusions on this point), studies covering additional components of the antioxidant system are necessary before ruling this possibility out.

### Oxidative stress as a constraint for reproduction

A constraining role of oxidative stress on life-history evolution has recently been proposed by [10]. This idea was illustrated for the immune system by [20] reporting that high pre-challenge systemic production of ROS is negatively related to the strength of subsequent cellular immune responses in male dragon lizards (*Ctenophorus pictus*). Our dataset was collected on a small mammal and supports the theory that oxidative stress plays a conserved constraining role on a closely fitness-related trait, reproduction. Indeed, we found that female mice with low pre-reproductive oxidative damage produced larger litters at birth compared to females with high pre-reproductive oxidative damage. The theory that oxidative stress has a constraining role on reproduction is also supported by data on clutch size and egg hatchability in a wild bird, the Alpine swift (*Apus melba*, [9]). Female swifts showing higher red cell membrane resistance to oxidative stress (measured shortly after egg laying) produce larger clutches, and eggs produced by these females were more likely to hatch [9]. Using a cross-fostering experiment where clutches were swapped between nests, the authors also showed that hatching failure was related to the production of low quality eggs by females with low resistance to oxidative stress rather than to inadequate parental care during incubation [9]. Our study suggests that oxidative stress has a constraining effect on only one part of the reproductive event: pre-reproductive oxidative damage was significantly related to litter size at birth but not to litter size at weaning. Our results imply that oxidative stress constrained the early investment in reproduction (i.e. conception and gestation) in female laboratory mice but did not constrain reproductive effort associated with rearing (i.e. lactation). Still, studies linking pre-reproductive oxidative balance to subsequent investment or success in reproduction are scarce, and the nature of the underlying mechanisms remains under investigated (see [11] for results from the medical literature, e.g. reduced sperm motility due to ROS). Hence, one suggestion is that early reproduction is probably affected more by the constraining effect of oxidative stress, whilst rearing is more likely to explain the oxidative cost of reproduction. Here, it is worth pointing that delayed costs of reproduction may further complicate the picture if past reproduction costs are constraining future reproductive success [21,22]. Experiments involving the manipulation of pre-reproductive oxidative stress are now required to validate the hypothesis of oxidative stress as a constraint for reproduction.

### Disentangling constraint and cost of oxidative stress on reproduction

Present knowledge on the relationships between reproduction and oxidative stress are complex and often contradictory, as summarized in Table 3 which lists studies reporting links between reproduction and four common markers of the oxidative balance: plasmatic antioxidant defences measured by the OXY test, plasmatic oxidative damage measured by the d-ROMs test, lipid peroxidation measured by MDA or TBARS tests, and resistance of red blood cell membranes to an oxidative burst (KRL test). Here, we argue that some of these contradictions could be resolved by considering the dual role (constraint and/or cost) of oxidative stress in the biology of reproduction. Indeed, a constraining role of oxidative stress on reproduction will be expressed through negative relationships between systemic (i.e. pre-reproductive) oxidative stress and reproduction, whereas the oxidative cost of reproduction will be revealed by post-reproductive increase in oxidative stress. It follows therefore that the relationships between reproduction and oxidative stress can be blurred by one prime factor, namely the timing chosen to sample animals because both the direction and the strength of these relationships are expected to vary over the course of reproduction. In agreement with this hypothesis, Table 3 shows that modifications of oxidative parameters in relation to reproduction seem to rely on whether animals were sampled before, during or after reproduction. Few studies report associations between reproduction and measures of the oxidative balance before or early in the reproductive phase [9,23-25], yet this is a pre-requisite to address the constraining role of oxidative stress. Interestingly, the association between reproduction and a given marker of oxidative stress in these studies often goes in the opposite direction to those described in studies where sampling occurred later in the reproductive phase (Table 3; present results). Table 3 also supports the idea that reproduction can incur oxidative costs and that costs are likely to increase with reproductive effort, indicating that sampling animals during reproduction (i.e. at the peak of metabolic demands, [26-28]) provides more proof of the effects of reproduction on the oxidative balance (10 of 14 studies, Table 3) than through sampling at the end or after reproduction (0 of 2 studies, Table 3). It suggests that the oxidative imbalance induced by reproduction may be transient [29] and could easily be missed if the sampling is wrongly timed. In this context, longitudinal design studies where animals are sampled before and during the course of reproduction ([7,30,31], present study, see also [32] for repeated measurement throughout life and long term oxidative consequences of reproduction) might provide a more powerful approach to detect an oxidative cost of reproduction than cross-sectional studies, as well as providing the opportunity to explore the constraining role of oxidative stress on reproduction (present study). Hence, studies on oxidative costs of reproduction should, ideally, control for the initial (pre-breeding) oxidative stress values by adding those values as covariate in the statistical models or by analysing changes in oxidative stress values. Finally, it is worth noting that experimental studies on costs of reproduction are generally based either on manipulation of reproductive status (i.e. comparisons between reproducing and non-reproducing individuals) or of reproductive effort (i.e. comparison between reduced and enlarged litter sizes). Those two experimental approaches however do not address the same reproductive costs *stricto sensu*. Manipulation of reproductive status allows testing for costs induced with decisions to reproduce or not, with reproducing individuals showing optimal investment into reproduction. In contrast, litter size manipulation is testing for costs induced by a deviation from optimal investment into reproduction. Because little is known on the relative importance of those different forms of reproductive costs, caution is needed in interpreting and comparing results of experiments of reproductive status *versus* effort.

**Table 3:** Review of existing literature on the relationships between reproduction and oxidative stress

		**Study type**	**Study design**	**Sampling time**	**Sample type**	**Sex**	**Relationship with reproduction**	**Reproduction trait**	**References**
**MDA or TBARS | Oxidative damage**									
Sprangue-Dawley rat	*Rattus norvegicus*	L	EXP_RS_	During	Lung	F	+	RS	[26]
Sprangue	*Rattus norvegicus*	L	EXP_RS_	During	Uterus	F	+	RS	[26]
Sprangue	*Rattus norvegicus*	L	EXP_RS_	During	Kidney	F	+	RS	[26]
Sprangue	*Rattus norvegicus*	L	EXP_RS_	During	Thymus	F	0	RS	[26]
Holtzman rat	*Rattus norvegicus*	L	EXP_RS_	During	Kidney	F	+	RS	[27]
Holtzman rat	*Rattus norvegicus*	L	EXP_RS_	During	Liver	F	+	RS	[27]
Red legged partridge	*Alectoris rufa*	C	COR	During	Erythrocyte	F/M	+/0	HS	[41]
Eastern chipmunks	*Tamias striatus*	W	COR	During	Plasma	F	+	BS	[13]
House mouse	*Mus musculus dimesticus*	L	EXP_RS_	During/ End	Liver	F	-	RS	[15]
Bank vole	*Myodes glareolus*	L	EXP_RS_	During/ End	Liver	F	0	RS	[16]
Bank vole	*Myodes glareolus*	L	EXP_RS_	During/ End	Kidney	F	0	RS	[16]
Bank vole	*Myodes glareolus*	L	EXP_RS_	During/ End	Heart	F	0	RS	[16]
Bank vole	*Myodes glareolus*	L	EXP_RS_	During/ End	Muscle	F	-	RS	[16]
Soay sheep	*Ovis Ovaries*	W	COR	End	Plasma	F	0	RS	[14]
**dROM | Oxidative**									
C57-Black6 maouse	*Mus Musculus*	L	COR	Before	Plasma	F	-	BS	Present Study
Great tit	*Parus Major*	W	COR	Before	Plasma	F	0	BS	[23]
Common starling	*Sturnus Vulgaran*	W	COR	Early	Plasma	F	-	CS	[23]
Tasmanian Spotted snow skink	*Niveoscincus ocellates*	W	COR	Early	Plasma	F	0	BS	[24]
Collared Flycatcher	*Ficedula albicollis*	W	COR	Early	Plasma	F	0	CS	[25]
Adélie penguins	*Pygoscelis adeliae*	W	COR	Early	Plasma	F/M	0/0	RE	[33]
*Eurasian kestrel*	*Falco tinnunculus*	W	COR	Early	Plasma	F/M	0/+	RS	[34]
Seychelles warbler	*Acrocephalus sechellensis*	W	COR	Δ [During-Before]	Plasma	F/M	0 in malaria infected birds	RS	[30]
Seychelles warbler	*Acrocephalus sechellensis*	W	COR	Δ [During-Before]	Plasma	F/M	-in malaria infected birds	RS	[30]
C57-Black 6 mouse	*Mus Musculus*	L	COR	Δ [During-Before]	Plasma	F	+	RS	Present study
C57-Black 6 mouse	*Mus Musculus*	L	COR	During /End	Plasma	F	+	BS	Present study
**OXY | Antioxidant**									
C57-Black mouse	*Mus musculus*	L	COR	Before	Plasma	F	0	BS	Present study
Great tit	*Parus major*	W	COR	Early	Plasma	F	-	CS	[23]
Common Starling	*Sturnus Vulgaris*	W	COR	Early	Plasma	F	+	CS	[23]
Tasmanian Spotted snow skink	*Niveoscinus ocellatus*	W	COR	Early	Plasma	F	0	BS	[24]
Collared Flycatcher	*Ficedula albicollis*	W	COR	Early	Plasma	F	0	CS	[25]
Adélie penguins	*Pygoscelis adeliae*	W	EXP_RE_	During	Plasma	F/M	+/+	RE	[33]
*Eurasian kestrel*	*Falco tinnunculus*	W	COR	During	Plasma	F/M	+/0	RS	[34]
Seychelles warbler	*Acrocephalus sechellensis*	W	COR	Δ [During-Before]	Plasma	F/M	-	RS	[30]
C57-Black 6 mouse	*Mus Musculus*	L	COR	Δ [During-Before]	Plasma	F	0	RS	Present study
C57-Black 6 mouse	*Mus Musculus*	L	COR	During /End	Plasma	F	0	RS	Present study
**KRL Â© | Resistance of red blood cell membranes to an oxidative burst**									
Alphine swift	*Apus melba*	W	COR	Early	Erythrocyte	F/M	+/0	HS	[9]
Alphine swift	*Apus melba*	W	COR	Early	Erythrocyte	F/M	+/0	CS	[9]
Great tit	*Parus major*	W	EXP_RE_	During	Erythrocyte	M	-	RE	[29]
Great tit	*Parus major*	W	EXP_RE_	During	Erythrocyte	F/M	-	RE	[28]
Zebra finch	*Taeniopygia gutata*	L	EXP_RE_	During	Erythrocyte	F/M	−/−	RE	[7]
Zebra finch	*Taeniopygia gutata*	L	COR	Δ [After-Before]	Erythrocyte	F/M	−/−	CS	[31]
Great tit	*Parus major*	W	EXP_RE_	Δ [After-Before]	Erythrocyte	M	0	RE	[29]

To examine the importance of sampling time in relation to reproduction, we restricted our review to four markers of oxidative stress with existing measurements at different times during the reproduction. Within each marker, studies are ordered by sampling time in relation to reproduction. *Study type* refers to Captive (C), Laboratory (L) and Wild (W) conditions. *Study design* refers to correlative (COR) *versus* experimental studies that either manipulated individual Reproductive Status (EXP_RS_) or Reproductive Effort (EXP_RE_). *Reproduction trait* refers to Clutch Size (CS), Hatchling Success (HS), Brood Size (BS), experimental manipulation of Reproductive Effort (RE), and comparison of Reproduction Status (RS; namely, comparing reproducing to non-reproducing animals or intra-individual variation during the course of reproduction). If separate measurements were reported for females and males (i.e. F / M), relationships with reproduction for each sex are separated by a /.

The choice of oxidative marker can also be a crucial element. Indeed, if we had based our interpretations on antioxidant defences alone, we would not have found correlative evidence for a constraining role of oxidative stress on reproduction or for an oxidative cost of reproduction. Although numerous methodological arguments could explain this lack of results, including the weak repeatability/accuracy of our antioxidant marker, small sample sizes and *ad libitum* access to a high-quality laboratory diet, our study backs up previous recommendations for the measurements of at least two components of the oxidative balance [12]. Yet, it is not always possible to assess more than one part of the oxidative balance, and in these circumstances we suggest that researchers should give priority to measuring oxidative damage. Indeed, oxidative damage is expected to be deleterious for the organism [5], whilst a decrease in antioxidant defences will only be costly if the production of ROS remains constant or increases during the same period. In other words, cost and constraint of oxidative stress on reproduction are more likely to be revealed by measurements of oxidative damage.

Finally, optimization of the oxidative balance (ROS production vs. antioxidant defences, and thereby damage) is likely to differ among species and between males and females within the same species. For example, long-lived species are expected to optimize survival at the expense of reproduction [1], which might be achieved via the up-regulation of antioxidant defences in order to prevent the accumulation of debilitating oxidative damage. This scenario is supported by data in Adélie penguins (*Pygoscelis adeliae*; [33]) and female Eurasian kestrels (*Falco tinnunculus*; [33,34]) where reproduction is associated with an increase in plasmatic antioxidants but not with changes in oxidative damage. The latter also shows that male kestrels did not up-regulate their antioxidant defences in response to reproduction, and in turn suffered from oxidative damage [34]. This is in conformity with Bateman’s principle, which describes greater selection on female than male survival.

## Conclusions

Early evolutionary biologists considered that animal responses were always optimal because adaptation inevitably results from the selection process. However, since the publishing of the corner-stone paper written by Gould and Lewontin [35], the key role of evolutionary constraints has emerged as “ biases on the production of variant phenotypes or limitations on phenotypic variability”, either due to structural or historical factors [36]. Our study highlights the constraining role of oxidative stress on reproduction [10], as previously outlined for other systems such as hormonal control [37]. This conclusion is further supported by recent findings in adult Florida scrub-jays (*Aphelocoma coerulescens*) where high levels of pre-breeding oxidative damage were associated with a decreased reproductive effort in males only [38]. Oxidative state is probably tightly linked to mitochondrial functioning, itself closely related to metabolic demands during reproduction, thereby giving a mechanistic basis for this relationship. It is worth noting that reproduction in mammals covers two very different phases, gestation and lactation, and we still know very little about how far these two phases contribute to shaping the oxidative cost of reproduction. Finally, looking for other possible oxidative constraints on any fitness-related trait, especially in free-living species, may help us to conclude how far oxidative stress plays an evolutionary role in shaping life-history trade-offs.

## Materials and methods

### General procedure

The study complied with the ‘Principles of Animal Care’ publication no.86-23, revised 1985 of the National Institute of Health, and with current legislation (L87-848) on animal experimentation in France. The study started with 23 primiparous adult female mice (C57 black 6) aged between 5 and 7 months from our animal husbandry unit. Females were housed in individual cages (40 × 25 × 15 cm) maintained at 25°C, on a 12 L : 12 D light cycle, and food (SAFE A03) and water were provided *ad libitum*. For mating purposes, one male was assigned randomly to each female and placed in the female’s cage for 7 days before being removed. Eighteen of the twenty-three mated females gave birth (thereafter named ‘successful females’). Five ‘unsuccessful females’, which did not show signs of pregnancy and did not produce pups, were used thereafter to assess changes in oxidative stress parameters independently of pups rearing over the time course of the study. Successful and unsuccessful females were of similar age (mean ± SE = 6.06 ± 0.21 *vs*. 5.80 ± 0.37; t-test, t = −0.58, df = 21, *p* = 0.57) and mass (22.9 ± 0.57 *vs*. 24.2 ± 0.89; t-test, t = −1.09, df = 21, *p* = 0.29). Although it is common for mice, and other rodents, that females are not all giving birth when mated, we cannot exclude that some of our unsuccessful females were unfertile (i.e. low-quality individuals). Nonetheless those unsuccessful females provide a valuable opportunity to investigate time-related change in oxidative markers in females involved in mating but not rearing pups. A week before pair formation, an initial blood sample (60 μL) was taken from all females (hereafter referred to as *before reproduction*). A second blood sample was taken 40 days after pair formation for both successful and unsuccessful females, which corresponded to the end of the lactation phase (i.e. 7 days before weaning) for successful females (hereafter referred as *after reproduction*). Blood was collected with a heparinised glass capillary tube from the submandibular vein. Plasma was recovered following centrifugation for 10 min at 3000 *g* and 4°C, and subsequently stored at −20°C up to three month before analysis according to manufacturer instructions.

### Oxidative stress measurements

The antioxidant barrier and the concentration of Reactive Oxygen Metabolites (ROMs) were measured using the OXY-Adsorbent (2 μL of plasma) and d-ROMs tests (5 μL of plasma, DIACRON INTERNATIONAL, s.r.l, Italy) following the manufacturer’s protocol (for detailed description of these tests, see [39]). The OXY adsorbent test was used to quantify the ability of the plasma antioxidant barrier to buffer massive oxidation through hypochlorous acid, while the d-ROMs test mostly measures hydroperoxydes as a marker of global early oxidative damage (principally on lipids and proteins). Antioxidant barrier is expressed as mM of HClO neutralised and d-ROMs as mg of H_2_O_2_ equivalent/dL. All measurements were run in duplicates and intra-individual variation was low (respectively 1.96 ± 0.34% for the OXY test and 2.76 ± 0.86% for the d-ROMs test). Measurements for the same individual before and after reproduction were run within the same laboratory session, and measurements of all the samples were divided in three laboratory sessions. Inter-session variations in the measurement (based on one sample repeated in all the session) were 4.52% for the OXY test and 5.31% for the d-ROMs test. Repeatability, i.e. the proportion of variability explained by the individual, was calculated following [40]. Both d-ROMs (ANOVA, F_1, 35_ = 26.41, *p* <0.001, r = 0.585) and Oxy-Adsorbent (ANOVA, F_1, 35_ = 4.90, *p* <0.034, r = 0.178) tests were shown to be repeatable over the study.

### Statistics

Changes in oxidative damage and antioxidant defences measured in the same individuals before and after reproduction were tested with generalized linear mixed-models (GLMM) where the reproductive status (reproductive success or not) and the reproductive period (before and after reproduction) were entered as two fixed factors and individual identity was used as a random factor. Post-hoc effects of reproductive status on oxidative balance were tested with paired t-tests. Constraint / cost of oxidative stress on reproduction was investigated by running ANCOVAs with litter size at weaning / at birth as dependent variables and OXY and d-ROMs measurements at weaning / prior to reproduction as co-variables, respectively. GLMM and ANCOVAs were fitted with a normal error distribution (SPSS 18.0). Analyses were two-tailed tests and p values ≤ 0.05. Means obtained from Mixed Models are Estimated Marginal Means and all means are quoted ± S.E.

## Competing interests

The authors declare that they have no competing interests.

## Authors’ contributions

AS and FC designed the study, AS and SR collected and analyzed the data. AS, PB and FC wrote the paper. SM, PB and FC took part in data interpretations. FC and PB share the seniorship of this article. All authors have read and approved the final version of the manuscript.
